# Optimizing Screening for Obstructive Sleep Apnea: Comparative Assessment of STOP and STOP-BANG Questionnaires in Croatia, Türkiye, and Greece

**DOI:** 10.3390/medicina62051002

**Published:** 2026-05-21

**Authors:** Ivana Pavlinac Dodig, Renata Pecotic, Natalija Ivkovic, Linda Lusic Kalcina, Özen K. Basoglu, Athanasia Pataka, Mehmet Sezai Tasbakan, Serapheim Kotoulas, Zoran Dogas

**Affiliations:** 1 Department of Neuroscience and Sleep Medicine Center, University of Split School of Medicine, 21000 Split, Croatia; ivana.pavlinac@mefst.hr (I.P.D.); nivkovic@gmail.com (N.I.); linda.lusic@mefst.hr (L.L.K.); zoran.dogas@mefst.hr (Z.D.); 2 Department of Respiratory Medicine, Ege University Faculty of Medicine, 35100 Izmir, Türkiye; ozenbasoglu@yahoo.com (Ö.K.B.); sezai72000@yahoo.com (M.S.T.); 3 Respiratory Failure Unit, G. Papanikolaou Hospital, Aristotle University of Thessaloniki, 57010 Thessaloniki, Greece; patakath@yahoo.gr; 4 Adult ICU, General Hospital of Thessaloniki “Ippokrateio”, 54642 Thessaloniki, Greece; akiskotoulas@hotmail.com

**Keywords:** obstructive sleep apnea, STOP, STOP-BANG, questionnaire, screening

## Abstract

*Background and Objectives*: Obstructive sleep apnea (OSA) is a common disorder associated with significant cardiovascular, metabolic, and neurocognitive consequences. The STOP and STOP-BANG questionnaires are widely used screening tools for identifying individuals at increased risk of OSA. However, their performance may vary across populations. This variability is due to demographic and anthropometric differences. We aimed to analyze the screening accuracy of the STOP and STOP-BANG questionnaires across three distinct Mediterranean populations: Croatia, Greece, and Türkiye. Additionally, we aimed to optimize and establish population-specific cut-off points for body mass index (BMI) and neck circumference (NC) in the questionnaires to enhance their screening accuracy. *Materials and Methods*: A total of 9102 patients who underwent polysomnography or polygraphy to evaluate suspected OSA were enrolled from: Split Sleep Medicine Centre (Croatia), Ege University Faculty of Medicine (Türkiye), and Thessaloniki G Papanikolaou Hospital Aristotle University (Greece). Patients completed the STOP and STOP-BANG questionnaires before sleep assessments. Sensitivity, specificity, and the area under the receiver operating characteristic (ROC) curve (AUC) were calculated to assess the screening properties. Additionally, optimized cut-offs for age, NC, and BMI were determined. *Results*: The highest AUC values were observed using the STOP-BANG ≥ 5 method, with AUC values of 0.712 for detecting any OSA (AHI ≥ 5/h), 0.684 for moderate or severe OSA (AHI ≥ 15/h), and 0.663 for severe OSA (AHI ≥ 30/h). For individual centers, the STOP-BANG ≥ 5 method performed best in Split, while the STOP ≥ 2 + NC method yielded the highest AUCs in Izmir and Thessaloniki for moderate and severe OSA. Optimized cut-off values for age, NC, and BMI improved sensitivity and specificity across all centers. *Conclusions*: This study highlights the need for population-specific considerations in the screening for OSA. Significant differences in demographics, anthropometrics, symptoms, and comorbidities across populations could impact the questionnaire’s screening accuracy. Adjusting age, NC, and BMI cut-off points optimizes the STOP-BANG questionnaire.

## 1. Introduction

Obstructive sleep apnea (OSA) is an important public health concern due to its increasing prevalence and the frequent appearance of comorbidities [1]. Given the limited availability of sleep studies in certified sleep medicine centers and the increasing workload of professionals and educated experts, screening tests are often used to assess OSA risk. Patients identified as high risk should be further evaluated with diagnostic procedures to objectively assess the presence of OSA. Some of the frequently used screening tools for OSA risk include the STOP and STOP-BANG questionnaires [2,3]. They both assess symptoms (snoring, tiredness, observed breathing cessations, and hypertension), while STOP-BANG also includes body mass index (BMI), age, sex, and neck circumference (NC) values. STOP-BANG is considered more comprehensive because it includes a detailed assessment of anthropometric and demographic data. However, previous studies have shown that it has higher sensitivity, but lower specificity than the STOP questionnaire, resulting in more false-positive results.

Dimensions and composition of the human body, including height, weight, waist-to-hip ratio, NC, and BMI, are of interest since they influence the risk for OSA. More specifically, with increasing BMI, there is an increase in risk for OSA due to a correlation between obesity and airway obstruction during sleep. Additionally, the upper airway and craniofacial features, as well as muscle function, can increase the likelihood of airway collapse during sleep. This is further accentuated by the involvement of NC involvement in risk assessment, as excessive fat in the neck region may narrow the airway during sleep, thus promoting OSA [4]. These anthropometric characteristics can vary significantly among different populations, due to the interplay among numerous factors that influence the anatomy and physiology of the individual and predisposing to OSA [5]. Genetic factors can interact with environmental influences and lifestyle habits, increasing the risk of obesity and OSA.

Mediterranean populations share certain similarities that indicate common inherited traits, reflecting historical migrations that have shaped them. In addition to genetic similarities, these people also share certain lifestyle habits and customs, particularly the Mediterranean diet, which is associated with numerous health benefits and often considered a key factor in longevity [6,7]. Although quite similar, we believe that even subtle differences among these populations might influence the screening accuracy of the tests that rely on anthropometric data.

Previous studies have shown that demographic and anthropometric characteristics can affect results and the likelihood of a positive screening questionnaire score [8,9]. Thus, adjusting the interpretation of demographic and anthropometric data, as well as the scoring methods of the questionnaires, could enhance their sensitivity and specificity among various patient populations, leading to better identification and selection of patients at risk for OSA [10].

Thus, this study aimed to comprehensively analyze the screening accuracy of the STOP and STOP-BANG questionnaires in terms of sensitivity and specificity across three distinct Mediterranean populations: Croatia, Greece, and Türkiye. Additionally, our secondary objective was to optimize the suggested cut-off points and establish population-specific cut-offs for BMI and NC in questionnaires to enhance their screening accuracy.

## 2. Materials and Methods

### 2.1. Study Population

This study was approved by the Biomedical Research Ethics Committee of the University of Split, School of Medicine for Croatia (approval number 2181-198-03-04-14-0027), Institutional Ethics Committee and Internal Review Board of Faculty of Medicine, Ege University for Türkiye (approval number 12-1.1/7), and for Thessaloniki (approval number 357/20160323). All study procedures were performed according to the ethical principles of the 1964 Declaration of Helsinki and its later amendments. All patients before the sleep assessment signed informed consent for participation.

The study was performed in the Split Sleep Medicine Centre (Split, Croatia), Ege University Faculty of Medicine Department of Respiratory Medicine (Izmir, Türkiye), and Aristotle University G Papanikolaou Hospital (Thessaloniki, Greece) during 2012–2023. A total of 9102 patients who underwent full-night polysomnography (PSG) or polygraphy (PG) to evaluate suspected OSA during the study period were enrolled. In Split, 1023 (32.9%) patients were assessed with PSG and 2085 (67.1%) with PG. In Izmir, a total of 3637 patients underwent PSG, while in Thessaloniki, all (2357) patients were evaluated with PG. This was a multicenter retrospective observational study conducted in tertiary sleep medicine centers in Croatia, Türkiye, and Greece.

### 2.2. Polysomnography

Full-night PSG/PG was performed and scored, and OSA was diagnosed according to the criteria of the American Academy of Sleep Medicine (AASM) Manual for Scoring Sleep and Associated Events and the European Sleep Research Society (ESRS) guidelines. In Split, Croatia, for full-night PG recording, either SOMNOcheck2 (Weinmann, Hamburg, Germany) or Alice Night One (Philips Respironics, Eindhoven, The Netherlands) was used, while for PSG, Alice 6 (Philips Respironics, Eindhoven, The Netherlands) was used. In Izmir, Türkiye, Alice 5 (Philips, Respironics, Pittsburgh, PA, USA) was used. In Thessaloniki, Greece, either SOMNOcheck2 (Weinmann, Hamburg, Germany) or Nox T3 (Nox medical, Iceland) was used. An apnea–hypopnea index (AHI) <5 events/h was considered no OSA, and data were grouped according to the established baseline AHI severity levels (mild ≥5 to <15 events/h, moderate ≥15 to <30 events/h and severe ≥30 events/h).

### 2.3. STOP and STOP-BANG Questionnaires

On the night of admission, before the sleep assessment, all patients filled out the STOP and STOP-BANG questionnaires. Both the STOP and the STOP-BANG questionnaires are easy-to-use tools for screening patients for OSA and have good reliability and internal consistency. The STOP questionnaire consists of 4 anamnestic questions: snoring (S), tiredness (T), observed breathing cessations (O), and arterial pressure (P); while BANG considers anthropometric and demographic data such as BMI (B), age (A), neck circumference (N), and gender (G). The STOP questionnaire indicates high OSA risk if there are 2 or more positive answers. However, there are various methods to interpret the STOP-BANG questionnaire: it is considered positive when the STOP (2 or more positive answers) is positive plus one additional component of the BANG section, or when there are 3 or more, or 5 or more positive answers in the STOP-BANG questionnaire [2,3].

### 2.4. Statistical Analysis

Data were analyzed using the Statistical Package for the Social Sciences (SPSS, version 20.0 for Windows) program. Continuous variables were reported as means ± standard deviations, while categorical variables as counts (percentages). Before analysis, the normality of data was assessed via the Shapiro–Wilk test.

One-way analysis of variance (ANOVA) was used to compare demographic, anthropometric, and sleep parameter data among three centers, followed by Tukey’s post hoc test to identify pairwise differences. Differences in symptom prevalence, comorbidities, and other categorical clinical characteristics were evaluated using the chi-square test. For the evaluation of screening performance and accuracy of the STOP and STOP-BANG questionnaires, receiver operating characteristic (ROC) curve analyses were used. Area under the curve (AUC) values were calculated for detecting OSA at various severity thresholds (e.g., AHI ≥ 5/h, AHI ≥ 15/h, and AHI ≥ 30/h). Additionally, sensitivity and specificity values were reported. The optimal cut-off values for age, NC, and BMI were derived and evaluated within the same dataset using the Youden index approach. Therefore, these findings should be considered exploratory and require external validation in independent cohorts.

Additional sex-stratified analyses were performed to evaluate the screening performance of the STOP and STOP-BANG questionnaires separately in male and female participants. Furthermore, analyses stratified by diagnostic modality (PSG vs. PG) were performed.

All statistical tests were two-tailed, and a *p* < 0.05 was considered statistically significant.

## 3. Results

### 3.1. Demographic and Anthropometric Data

A total of 9102 patients (69.5% male) from Greece, Türkiye, and Croatia were included. Specifically, patients from Thessaloniki, Greece (2357; 25.9%) underwent full-night PG, while those from Izmir, Türkiye (3637; 40%) and Split, Croatia (3108; 34.1%) were assessed using PG (67.1%) or PSG (32.9%). Notably, there were no differences in sex distribution among centers. Thessaloniki patients had the highest body weight (98.8 ± 22.5 kg) and BMI (33.4 ± 7.1 kg/m^2^), while Split patients were the tallest (1.77 ± 0.10 m) (all *p* < 0.001). Further demographic and anthropometric data are presented in Table 1.

### 3.2. Clinical Characteristics

Izmir patients most frequently reported snoring (98.2%), daytime tiredness (79.5%), and observed breathing cessations (81.4%), while Split patients had the highest prevalence of arterial hypertension (45.9%). Diabetes, depression, and gastroesophageal reflux disease were most common in Izmir (Table 2).

### 3.3. Sleep Parameters

The average AHI was highest among patients from Izmir (37.7 ± 28.8/h), compared to those from Thessaloniki (32.7 ± 24.8/h) and Split (25.3 ± 22.4/h) (*p* < 0.001). The mean blood oxygen saturation of patients from Thessaloniki (91.9 ± 3.4%) was lower than that of the patients from Izmir (92.7 ± 14.4%) and Split (94.2 ± 3.3%) (*p* < 0.001) (Table 2).

### 3.4. Screening Performance of STOP and STOP-BANG Questionnaires Across Different Populations

Table 3 presents the sensitivity, specificity, and AUC values of the STOP and different STOP-BANG assessment methods. The STOP-BANG ≥ 5 method had the best screening properties overall (AUC for OSA: 0.712, for moderate or severe OSA: 0.684, for severe OSA: 0.663).

In Thessaloniki, STOP-BANG ≥ 3 was most effective for OSA, while STOP ≥ 2 + NC was best for moderate to severe OSA. In Izmir, STOP-BANG ≥ 5 was optimal for detecting OSA and moderate or severe OSA, while STOP ≥ 2 + NC was best for severe OSA. In Split, STOP-BANG ≥ 5 was the best for all categories (AUCs: 0.720 for OSA, 0.707 for moderate/severe OSA, 0.701 for severe OSA).

### 3.5. Impact of Optimized Cut-Offs on Screening Accuracy of the STOP-BANG Questionnaire

Cut-off points for age, NC, and BMI were optimized to improve the screening properties of the STOP-BANG questionnaire (Table 4). Age cut-offs ranged from 45.5 years (Split) to 54.5 years (Izmir), NC ranged from 40.5 cm (Thessaloniki) to 40.8 cm (Izmir and Split), and BMI ranged from 26.4 kg/m^2^ (Split) to 30.7 kg/m^2^ (Thessaloniki).

Finally, when these optimized cut-off values were applied, changes in the screening accuracy of the STOP-BANG questionnaire further increased sensitivity and decreased specificity, and reflected in the changes in AUC values of the STOP-BANG questionnaire in each population (Table 5).

Sex-stratified analyses showed similar overall trends in screening performance in both sexes, with generally higher sensitivity observed in males and relatively higher specificity in females. In both groups, the STOP-BANG ≥ 5 approach performed best (Table S1). For additional analyses stratified by PSG and PG, the STOP and STOP-BANG questionnaires showed similar screening performance in both modalities, with no substantial differences in results (Table S2). ROC curve (Supplementary Figure S1) illustrate and compare AUC values for the STOP and STOP-BANG questionnaire methods in detecting OSA at various AHI thresholds across the studied populations.

## 4. Discussion

A large cohort of 9102 patients presenting to sleep medicine centers for OSA diagnostics from three Mediterranean countries, Greece, Türkiye, and Croatia, was included in this study. The studied populations share many similarities. This might be due to their shared Mediterranean background, reflected in their lifestyle and genetic traits linked to health benefits, such as cardiovascular health and longevity. Populations with OSA in Mediterranean countries, such as Greece, Türkiye, and Croatia, differ from other OSA populations due to specific demographic, anthropometric, and health factors. However, despite these common characteristics, notable differences among these populations may have influenced the risk factors, presentation, and OSA severity. These differences likely include variations in anthropometric measures, such as obesity and BMI, as well as the prevalence of comorbidities like hypertension, diabetes, and depression. Our study revealed that patients in Greece had higher BMIs, which are associated with more severe OSA. Higher rates of comorbidities such as diabetes and GERD were observed in Türkiye. These factors appeared to influence both the presentation and severity of OSA. One might argue that these differences could influence the results of OSA screening tests. This is particularly important since inconsistencies in epidemiological studies have previously been linked to racial and/or ethnic background, study design, age, BMI, and sex [11]. It is important to note that the optimal cut-off points for OSA risk assessment using the STOP-BANG questionnaire vary across populations. This emphasizes the need to adapt diagnostic approaches to the specific characteristics of each region.

Our findings support this view, as different STOP-BANG assessment methods yielded varying screening accuracy across the three studied populations. Moreover, the distinct anthropometric characteristics of each region influenced the optimal cut-off values. These observations highlight the importance of considering racial, ethnic, anthropometric, and craniofacial variability when interpreting OSA screening questionnaires and selecting appropriate diagnostic thresholds. Population-specific adaptations in screening and diagnosis may therefore improve the accuracy of OSA detection. Nevertheless, these findings should be validated in larger multicenter and multiethnic cohorts before broader implementation in routine clinical practice.

In terms of OSA screening, the STOP and STOP-BANG questionnaires are reliable and widely used tools for OSA risk assessment [12,13,14]. Still, the assessment method for interpreting the STOP-BANG questionnaire, which results in the best screening accuracy across different populations, is questionable [15,16]. Results from the present study revealed that the best screening properties with the highest AUC values for detecting mild, moderate, and severe OSA had STOP-BANG ≥ 5 method in the total sample, but not when three populations were analyzed separately. The differences in predictive values, such as the lower specificity of the STOP-BANG questionnaire in Thessaloniki and Izmir compared to Split, can be attributed to several factors. First, the anthropometric characteristics of the populations vary, with patients in Thessaloniki and Izmir having higher BMI and NC, which could lead to higher screening rates even in individuals without severe OSA. Additionally, differences in the prevalence of comorbidities such as hypertension may also influence the specificity of the screening tool. Variations in the cut-off points used for the STOP-BANG questionnaire across center further reflect these population differences, indicating that regional factors influence the screening tool’s performance. Thus, the specific characteristics of each population, including anthropometric and demographic factors, and comorbidities, likely contribute to the observed discrepancies in predictive values. In Thessaloniki, the STOP-BANG ≥ 3 method was most effective for detecting OSA, while the STOP ≥ 2 + NC method performed best for detecting moderate and severe OSA. The same method performed the best for detecting patients with severe OSA in Izmir, while STOP-BANG ≥ 5 method was better in screening patients with moderate OSA. In Split, STOP-BANG ≥ 5 method was the best assessment method in all OSA severity groups. These results suggest that using different assessment methods for the STOP-BANG questionnaire across populations is important to enhance screening accuracy, especially given anthropometric differences between populations. Our findings support the idea that although STOP-BANG is a reliable tool, its interpretation method needs to be adjusted to optimize its sensitivity and specificity in different populations.

Although all studied populations belonged to the Mediterranean region and shared some genetic and lifestyle traits, differences in anthropometric measures such as body weight, height, and BMI were still evident across three populations, possibly reflecting different OSA severity reported in the included patients. Patients from Thessaloniki exhibited the highest body weight and BMI, factors known to contribute to the risk and severity of OSA [17,18]. In particular, the assessment of OSA risk might be influenced by the differences in the prevalence of obesity, a well-established risk factor for OSA, across these cohorts [11]. The emerging threats of obesity, along with an aging population, significantly contribute to the global burden of OSA and impact the health of entire populations. With the persistent obesity epidemic, a rise in prevalence is imminent in countries related to the Mediterranean region since recent evidence supports an increased prevalence of obesity and metabolic syndrome in OSA patients from Mediterranean countries such as Greece, Spain, and Italy [19].

Furthermore, when considering the application of the STOP-BANG questionnaire across different geographic populations with varying anthropometry, it is essential to recognize that anthropometric factors, such as body weight and NC, may vary among diverse ethnic groups [20,21,22]. This recognition is particularly crucial since some of these factors are integral to the questionnaire itself. Thus, to further improve the STOP-BANG questionnaire’s screening accuracy, the cut-off points for age, NC, and BMI were optimized in each studied population. The optimal cut-off values varied between populations and reflected demographic and anthropometric differences. For instance, Thessaloniki had the highest optimal age cut-off. In contrast, Split had the lowest, suggesting that OSA risk in these populations increases at different ages. Similarly, NC cut-off points were slightly higher in Izmir and Split compared to Thessaloniki. This highlights the importance of tailoring screening criteria to population-specific anthropometric characteristics. The optimized BMI cut-off values were also lower in Split. This finding is consistent with the lower average BMI in this population and further supports the need for regional adaptations of screening tools to enhance their accuracy. One might conclude that it is important to adjust risk thresholds to reflect the anthropometric characteristics specific to a particular geographic population.

The optimized thresholds identified in this study should not be interpreted as strict center-specific cut-offs, but rather as population-specific estimates reflecting demographic and anthropometric differences among the Mediterranean populations studied. Their clinical application would require validation in independent regional or national cohorts before routine implementation. These findings primarily support the concept that adaptation of STOP-BANG criteria to local population characteristics may improve screening performance.

Importantly, the STOP-BANG questionnaire is designed as a screening tool with the primary goal of maximizing sensitivity to identify patients at increased risk for OSA who require further diagnostic evaluation. Consequently, lower specificity at certain thresholds should not necessarily be interpreted as a limitation, but rather as an expected feature of an effective screening strategy. In routine clinical practice, STOP-BANG results should always be interpreted in combination with clinical symptoms, daytime sleepiness, comorbidities, anthropometric characteristics, and physician’s clinical judgment. Therefore, the optimized cut-off values proposed in this study are not intended as standalone diagnostic thresholds, but rather as tools to improve risk stratification and prioritize PSG or PG assessment across different populations.

Consistent with previous literature, the STOP-BANG questionnaire demonstrated higher sensitivity but lower specificity compared with the STOP questionnaire alone. Although this may increase the number of false-positive results, such a characteristic is expected in screening tools designed to minimize missed cases of suspected OSA. Therefore, lower specificity should be interpreted in the context of the questionnaire’s intended screening purpose rather than as a limitation in itself. The optimized cut-off values proposed in this study may improve practical risk stratification and prioritization for further sleep testing, particularly in clinical settings with limited diagnostic resources.

In the present study, high rates of upper airway symptoms such as snoring and observed breathing cessations, combined with daytime tiredness, were reported in all populations studied. Patients from Izmir had the highest average AHI values and highest rates of reported symptoms, emphasizing the association between clinical symptoms and OSA severity. The association between higher AHI values, oxygen desaturation, and increased symptom burden is supported by previous findings demonstrating that oxygen desaturation index and AHI are important predictors of daytime sleepiness in patients with OSA [23]. It has been reported that OSA is associated with many comorbid disorders affecting the cardiovascular, respiratory, metabolic, and central nervous system, leading to adverse health outcomes [11]. Differences in the prevalence of comorbidities included in the STOP-BANG questionnaire, particularly hypertension, may influence the estimated OSA risk and screening performance across populations. Such variability may reflect differences in population health characteristics, healthcare systems, referral patterns, and diagnostic practices, further supporting the need for cautious interpretation of screening thresholds across different clinical settings. The higher prevalence of comorbidities such as diabetes, asthma, and GERD in Izmir further supports the notion that the OSA severity might be associated with unfavorable health outcomes [24,25]. However, despite having the lowest average AHI, patients from Split reported the highest prevalence of comorbid arterial hypertension, an established risk factor for cardiovascular complications in OSA patients [26]. The results of our study indicate that, although all studied populations belong to the Mediterranean region, some inter-ethnic factors may contribute to observed differences in susceptibility to comorbidities and affect the accuracy of the STOP-BANG questionnaire’s screening properties. Thus, further optimization and tailoring of screening interpretation may help better identify patients with a higher OSA risk who require prompt care [11].

From a clinical perspective, population-specific optimization of STOP-BANG thresholds may improve practical risk stratification in patients with suspected OSA and support more efficient prioritization for diagnostic sleep testing. Such an approach may be particularly valuable in healthcare systems with limited access to PSG or PG, where effective screening strategies are essential to reduce diagnostic delays and optimizing resource utilization. Future studies should focus on prospective multicenter validation of the proposed cut-off values in independent and more diverse populations, including community-based cohorts and broader multiethnic datasets. Additional research is needed to determine whether population-specific adaptations of the STOP-BANG questionnaire may improve clinical risk stratification and optimize referral pathways for PSG or PG testing in different healthcare settings.

The main strength of our study is that it is one of the largest conducted in university hospitals across three countries, in which demographic and anthropometric characteristics, detailed medical history, and sleep test (PSG/PG) data were available for all patients. However, including patients from three distinct sleep centers located in different geographic regions introduces potential limitations and biases in the patient populations studied, patient selection, and the clinical protocols used. The inclusion of patients from single tertiary sleep centers in each country may have introduced regional and institutional selection bias, potentially limiting the generalizability of the findings to wider national populations. Additionally, because the study population consisted of patients referred for sleep evaluation with suspected OSA, the cohort had a relatively high pretest probability of disease. This may have influenced the estimated screening performance of the STOP and STOP-BANG questionnaires and limits direct extrapolation of the results to community-based or general population screening settings. Because the prevalence of individual STOP-BANG components may vary between populations, the findings of the present study should be generalized cautiously to populations with different demographic, anthropometric, or comorbidity profiles. Another limitation relates to the use of self-reported questionnaires, which are subject to recall bias and subjective interpretation of symptoms, potentially affecting the estimated sensitivity and specificity of the screening tools. We acknowledge that the patients included in our study were diagnosed using two different methods, PG and PSG. The use of both methods, as well as different recording devices across centers, may have influenced OSA classification and the estimated screening performance of the questionnaires. Although all studies were performed and scored according to established AASM and ESRS criteria, methodological variability between centers and diagnostic modalities cannot be fully excluded. However, both methods are valid diagnostic tools for cases with a high clinical suspicion of uncomplicated OSA. The choice between these methods depends on the clinical setting and available resources. Additionally, the optimized cut-off values were derived and tested in the same study population, potentially leading to overfitting and limiting the external validity of these thresholds. Future studies should validate these cut-off points in independent populations. Another limitation is that this study evaluated only the STOP and STOP-BANG questionnaires and did not compare their performance with other established OSA screening tools, such as the Berlin Questionnaire, Epworth Sleepiness Scale, NoSAS, or GOAL questionnaire. Therefore, the present findings should be interpreted within the context of these specific screening instruments and do not allow conclusions regarding their comparative superiority over other available tools.

Thus, the conclusions of the present study should be interpreted within the context of a retrospective observational design and clinically referred sleep clinic populations. This limits direct extrapolation to community-based populations or causal interpretation of the observed associations. Future studies should aim to validate these findings in prospective multicenter cohorts. These cohorts should include community-based populations and independent external validation datasets. Additional studies involving broader multiethnic populations may further clarify the influence of anthropometric and demographic variability on the performance of OSA screening questionnaires. Such research can help define clinically applicable population-specific thresholds.

## 5. Conclusions

In conclusion, these findings suggest that the universal approach to OSA screening is not optimal, emphasizing the need for population-specific considerations, even when common lifestyle factors are shared. Significant differences in demographics, anthropometric measures, symptom prevalence, and comorbidities across populations might impact the accuracy of screening tools, such as the STOP-BANG questionnaire. Rather than supporting a universal ‘one-size-fits-all’ approach, our findings suggest that regional or population-specific adaptations of STOP-BANG thresholds may improve OSA screening accuracy in clinical practice. Our findings suggest that adapting STOP-BANG interpretation to population-specific characteristics may improve the clinical utility of OSA screening, contributing to earlier identification of high-risk patients, and potentially improved long-term patient outcomes.

Therefore, it is crucial to adapt the interpretation of this questionnaire to fit the unique characteristics of each population, including anthropometrics, obesity rates, or comorbidities. Finally, early detection of high-risk patients is essential not only for improving individual health outcomes but also for reducing the economic burden on national healthcare systems, leading to better long-term outcomes and more cost-effective management of OSA across different populations. Further large-scale prospective studies are warranted to externally validate the proposed thresholds and to explore their integration into comprehensive OSA screening strategies combining questionnaires, clinical characteristics, anthropometric parameters, and additional screening tools.

## Figures and Tables

**Table 1:** Demographic and anthropometric data of the study population.

	Overall N = 9102	Thessaloniki, Greece N = 2357 (25.9%)	Izmir, Türkiye N = 3637 (40%)	Split, Croatia N = 3108 (34.1%)	*p*
Age	53.82 ± 13.03	57.22 ± 13.07	51.21 ± 12.02	54.30 ± 13.47	<0.001 ^1^
Sex					
Male, N (%)	6322 (69.5)	1683 (71.4)	2496 (68.6)	2143 (69)	0.056 ^2^
Female, N (%)	2780 (30.5)	674 (28.6)	1141 (31.4)	965 (31)	
Weight (kg)	94.6 ± 20.7	98.8 ± 22.5	92.4 ± 18.8	94.1 ± 20.9	<0.001 ^1^
Height (m)	1.72 ± 0.10	1.72 ± 0.09	1.69 ± 0.10	1.77 ± 0.10	<0.001 ^1^
Body mass index (kg/m^2^)	31.9 ± 6.7	33.4 ± 7.1	32.6 ± 6.8	29.8 ± 5.8	<0.001 ^1^
Neck circumference (cm)	41.5 ± 4.8	41.5 ± 5.3	41.4 ± 4.0	41.5 ± 5.2	0.842 ^1^

^1^ One-way ANOVA; ^2^ Chi-square test. Sex data are shown as absolute and relative frequencies, all other data are shown as means ± standard deviations.

**Table 2:** Clinical characteristics and sleep parameters of the study population.

	Overall N = 9102	Thessaloniki, Greece N = 2357 (25.9%)	Izmir, Türkiye N = 3637 (40%)	Split, Croatia N = 3108 (34.1%)		*p* ^1^
**Clinical characteristics**						
Snoring						
Yes, N (%)	8109 (89.7)	2247 (95.3)	3573 (98.2)	2289 (75.2)		<0.001
Tiredness						
Yes, N (%)	6778 (74.8)	1588 (67.4)	2891 (79.5)	2299 (75.0)		<0.001
Observed breathing cessations						
Yes, N (%)	6661 (73.9)	1783 (75.6)	2960 (81.4)	1918 (63.5)		<0.001
Arterial hypertension						
Yes, N (%)	3863 (42.4)	980 (41.6)	1455 (40.0)	1428 (45.9)		<0.001
Diabetes mellitus						
Yes, N (%)	1740 (19.2)	382 (16.2)	893 (24.6)	465 (15.1)		<0.001
Asthma						
Yes, N (%)	1382 (15.2)	61 (2.6)	958 (26.3)	363 (11.8)		<0.001
Depression						
Yes, N (%)	408 (6.1)	0 ^2^	210 (5.8)	198 (6.4)		<0.001
GERD						
Yes, N (%)	2078 (22.9)	70 (3)	1116 (30.7)	892 (29.1)		<0.001
**Sleep parameters**						
Type of sleep test						
PSG, N (%)	4660 (51.2)	0 (0)	3637 (100)	1023 (32.9)		<0.001
PG, N (%)	4442 (48.8)	2357 (100)	0 (0)	2085 (67.1)		
		PG	PSG	PG	PSG	
OSA severity						
No OSA, N (%)	1065 (11.7)	318 (13.5)	279 (7.7)	258 (12.4)	210 (20.6)	<0.001
Mild OSA, N (%)	1940 (21.3)	353 (15)	679 (18.7)	651 (31.2)	257 (25.1)	
Moderate OSA, N (%)	1996 (21.9)	566 (24)	845 (23.2)	412 (19.8)	173 (16.9)	
Severe OSA, N (%)	4101 (45.1)	1120 (47.5)	1834 (50.4)	764 (36.6)	383 (37.4)	
AHI (events/h)	32.2 ± 26.3	32.7 ± 24.8	37.7 ± 28.8	25.3 ± 22.4		<0.001
Mean O_2_ saturation (%)	93.0 ± 9.5	91.9 ± 3.4	92.7 ± 14.4	94.2 ± 3.3		<0.001
Lowest O_2_ saturation (%)	79.4 ± 11.2	78.9 ± 9.8	78.9 ± 12.2	80.5 ± 11.0		<0.001

^1^ Chi-square test; ^2^ Missing data. All data are shown as absolute and relative frequencies. Abbreviations: AHI, apnea-hypopnea index; GERD, gastroesophageal reflux disease; OSA, obstructive sleep apnea.

**Table 3:** Sensitivity, specificity, and AUC values of STOP and various STOP-BANG assessment methods in the study population.

	Overall			Thessaloniki, Greece			Izmir, Türkiye			Split, Croatia		
AHI	≥5	≥15	≥30	≥5	≥15	≥30	≥5	≥15	≥30	≥5	≥15	≥30
STOP ≥ 2												
Sensitivity	92.7	94.9	96.5	93.0	94.7	96.1	97.4	97.9	98.8	86.5	90.6	93.3
Specificity	29.1	19.5	15.0	23.6	18.9	14.0	10.0	6.0	5.1	44.6	29.3	24.8
AUC	0.609	0.572	0.558	0.583	0.568	0.550	0.537	0.520	0.520	0.655	0.599	0.591
STOP ≥ 2 + BMI												
Sensitivity	23.3	27.5	32.1	37.1	41.9	49.2	30.0	32.6	37.4	4.1	5.5	6.9
Specificity	92.1	90.6	87.1	87.1	86.4	80.1	86.0	81.7	79.9	99.3	98.9	98.4
AUC	0.577	0.590	0.596	0.621	0.641	0.646	0.580	0.571	0.587	0.517	0.522	0.526
STOP ≥ 2 + Age												
Sensitivity	59.4	62.7	63.4	67.5	69.8	69.6	53.9	55.6	56.2	60.3	66.8	68.9
Specificity	69.8	57.6	50.0	59.7	51.1	41.4	73.5	59.0	52.7	74.6	59.8	53.0
AUC	0.646	0.602	0.567	0.636	0.605	0.556	0.637	0.573	0.545	0.674	0.633	0.609
STOP ≥ 2 + Neck												
Sensitivity	58.8	65.1	71.8	59.4	64.8	70.4	61.3	65.7	72.5	55.1	64.5	72.0
Specificity	79.0	67.5	59.9	80.8	73.3	60.9	67.4	59.5	54.5	84.8	70.2	64.2
AUC	0.689	0.663	0.658	0.701	0.692	0.657	0.643	0.626	0.635	0.700	0.674	0.681
STOP ≥ 2 + Sex												
Sensitivity	66.6	71.1	75.2	68.2	70.5	73.7	68.2	71.1	74.7	63.2	71.6	77.4
Specificity	64.0	53.4	47.0	56.9	49.5	43.2	54.5	46.5	41.8	74.6	60.2	54.1
AUC	0.652	0.622	0.611	0.626	0.600	0.584	0.613	0.588	0.582	0.689	0.659	0.657
STOP-BANG ≥ 3												
Sensitivity	96.2	98.4	99.3	97.7	98.6	99.6	98.2	98.8	99.6	92.5	97.5	98.7
Specificity	29.3	17.2	11.7	20.4	13.3	8.6	12.9	6.6	4.9	45.4	26.5	20.0
AUC	0.628	0.578	0.555	0.721	0.560	0.541	0.556	0.527	0.522	0.690	0.620	0.594
STOP-BANG ≥ 5												
Sensitivity	67.2	74.3	80.1	72.5	77.2	81.5	70.3	74.9	80.7	59.2	70.8	77.9
Specificity	75.1	62.3	52.4	71.7	60.1	47.0	63.1	52.2	45.4	84.8	70.5	62.2
AUC	0.712	0.684	0.663	0.591	0.687	0.643	0.667	0.636	0.630	0.720	0.707	0.701

Abbreviations: AUC, area under the curve; AHI, apnea–hypopnea index; BMI, body mass index.

**Table 4:** Cut-off points defined based on the maximum level of sensitivity and specificity (Youden index approach) for the detection of OSA (AHI ≥ 5 events/h).

		Cut-Off	Sensitivity	Specificity
Thessaloniki, Greece	Age (years)	47.21	0.789	0.437
Izmir, Türkiye	Age (years)	54.50	0.426	0.832
Split, Croatia	Age (years)	45.50	0.807	0.529
Thessaloniki, Greece	Neck (cm)	40.50	0.631	0.764
Izmir, Türkiye	Neck (cm)	40.75	0.620	0.670
Split, Croatia	Neck (cm)	40.75	0.642	0.771
Thessaloniki, Greece	BMI (kg/m^2^)	30.71	0.644	0.720
Izmir, Türkiye	BMI (kg/m^2^)	29.74	0.636	0.609
Split, Croatia	BMI (kg/m^2^)	26.41	0.779	0.611

Abbreviations: AHI, apnea–hypopnea index; BMI, body mass index; OSA, obstructive sleep apnea.

**Table 5:** Differences in the sensitivity, specificity, and AUC values of the STOP-BANG questionnaire with the use of previous and newly optimized cut-off points for body mass index, neck circumference, and age in the study population.

		Thessaloniki, Greece			Izmir, Türkiye			Split, Croatia		
	AHI	≥5	≥15	≥30	≥5	≥15	≥30	≥5	≥15	≥30
STOP-BANG ≥ 3 previous(^p^) vs. new(^n^) cut-off points	Sensitivity ^p^	97.7	98.6	99.6	98.2	98.8	99.6	92.5	97.5	98.7
	Specificity ^p^	20.4	13.3	8.6	12.9	6.6	4.9	45.4	26.5	20
	AUC ^p^	0.721	0.560	0.541	0.556	0.527	0.522	0.690	0.620	0.594
	Sensitivity ^n^	98.6	99.2	99.7	98.2	98.9	99.6	98.2	98.9	99.6
	Specificity ^n^	19.2	11.4	7.0	12.2	6.8	4.8	12.2	6.8	4.8
	AUC ^n^	0.589	0.553	0.534	0.552	0.528	0.522	0.659	0.583	0.563
STOP-BANG ≥ 5 previous(^p^) vs. new(^n^) cut-off points	Sensitivity ^p^	72.5	77.2	81.5	70.3	74.9	80.7	59.2	70.8	77.9
	Specificity ^p^	71.7	60.1	47	63.1	52.2	45.4	84.8	70.5	62.2
	AUC ^p^	0.591	0.687	0.643	0.667	0.636	0.630	0.720	0.707	0.701
	Sensitivity ^n^	79.3	83.8	87.6	73.0	77.2	82.9	73.0	77.2	82.9
	Specificity ^n^	66.7	53.8	40.0	60.2	48.2	42.1	60.2	48.2	42.1
	AUC ^n^	0.730	0.688	0.638	0.666	0.627	0.625	0.759	0.691	0.677

Abbreviations: AUC, area under the curve; AHI, apnea–hypopnea index.

## Data Availability

The data presented in this study are available on reasonable request from the corresponding author. The data are not publicly available due to privacy and ethical restrictions involving human participants.

## Supplementary Materials

The following supporting information can be downloaded at: https://www.mdpi.com/article/10.3390/medicina62051002/s1, Figure S1: Receiver operating characteristic (ROC) curve figures illustrating the area under the curve (AUC) values for the STOP and STOP-BANG questionnaire approaches, presenting the comparative screening performance for detecting obstructive sleep apnea at different apnea-hypopnea index (AHI) thresholds across the studied populations.; Table S1: Data in different centers stratified by gender; Table S2: Full data stratified by device recording type.

## Abbreviations

The following abbreviations are used in this manuscript:

OSA	Obstructive sleep apnea
BMI	Body mass index
NC	Neck circumference
PSG	Polysomnography
PG	Polygraphy
AHI	Apnea–Hypopnea Index
GERD	Gastroesophageal reflux disease
AUC	Area under the curve
ROC	Receiver operating characteristic
ANOVA	Analysis of variance
