# Supplementary material for: Optimizing Screening for Obstructive Sleep Apnea: Comparative Assessment of STOP and STOP-BANG Questionnaires in Croatia, Türkiye, and Greece
Source: Medicina (Kaunas). 2026 May 21;62(5):1002. doi: 10.3390/medicina62051002 (PMC13208978; doi:10.3390/medicina62051002)
Supplement: Supplementary file 1 [file medicina-62-01002-s001.zip › medicina-4317204-supplementary.pdf]

Table 1. Data in different centers stratified by gender

			**Thessaloniki, Greece**			**Izmir, Türkiye**			**Split, Croatia**		
		**AHI**	**≥5**	**≥15**	**≥30**	**≥5**	**≥15**	**≥30**	**≥5**	**≥15**	**≥30**
**MALE**	**STOP-BANG ≥3 previous(^{p}) vs. new(^{n}) cut-off points**	**Sensitivity^p**	98.8	99.52	100	99.45	99.69	99.86	92.54	97.46	98.69
		**Specificity^p**	13.19	8.45	5.11	1.46	1.64	1.17	45.43	26.54	20.03
		**AUC^p**	0.560	0.540	0.526	0.505	0.507	0.547	0.620	0.559	0.541
		**Sensitivity^n**	99.27	99.68	100	99.41	99.69	99.86	96.38	98.99	99.62
		**Specificity^n**	13.19	7.31	4.27	1.46	1.82	1.26	35.5	17.7	12.92
		**AUC^n**	0.562	0.672	0.521	0.504	0.508	0.546	0.609	0.546	0.531
	**STOP-BANG ≥5 previous(^{p}) vs. new(^{n}) cut-off points**	**Sensitivity^p**	79.48	83.37	86.53	78.76	82.34	86.48	59.15	70.78	77.94
		**Specificity^p**	64.29	50.7	37.59	45.99	40.15	33.87	84.78	70.45	62.24
		**AUC^p**	0.719	0.535	0.621	0.624	0.612	0.632	0.712	0.668	0.653
		**Sensitivity^n**	85.39	89.18	91.87	81.01	84.14	88.5	78.02	87.06	92.7
		**Specificity^n**	59.89	45.28	31.46	45.99	36.86	31.63	73.75	51.17	42.73
		**AUC^n**	0.726	0.672	0.617	0.635	0.605	0.622	0.725	0.640	0.627
**FEMALE**	**STOP-BANG ≥3 previous(^{p}) vs. new(^{n}) cut-off points**	**Sensitivity^p**	94.61	96.04	98.07	95.3	96.29	98.65	81.19	92.78	95.9
		**Specificity^p**	30.15	21.63	15.66	23.94	13.08	10.76	60.58	42.38	35.63
		**AUC^p**	0.624	0.588	0.569	0.596	0.547	0.547	0.715	0.673	0.655
		**Sensitivity^n**	96.65	97.67	98.84	95.3	96.7	98.65	90.97	97.27	99.44
		**Specificity^n**	27.21	18.37	12.53	22.54	13.32	10.47	46.63	28.08	22.99
		**AUC^n**	0.619	0.580	0.557	0.589	0.550	0.546	0.688	0.627	0.612
	**STOP-BANG ≥5 previous(^{p}) vs. new(^{n}) cut-off points**	**Sensitivity^p**	53.16	58.97	64.86	50.25	54.95	62.61	31.26	45.83	56.92
		**Specificity^p**	81.62	76.33	65.54	79.58	68.28	63.7	95.44	88.08	83.62
		**AUC^p**	0.674	0.677	0.652	0.649	0.616	0.632	0.643	0.670	0.706
		**Sensitivity^n**	62.45	68.07	73.36	54.15	58.52	65.54	56.07	70.3	78.33
		**Specificity^n**	75.74	68.57	56.87	73.94	63.2	58.82	87.5	70.38	63.43
		**AUC^n**	0.691	0.683	0.651	0.640	0.609	0.622	0.718	0.703	0.709

Abbreviations: AHI, apnea-hypopnea index; AUC, *area under the curve*

Table 2. Full data stratified by device recording type

	**AHI**	**≥5**		**≥15**		**≥30**	
	**Device**	**PSG**	**PG**	**PSG**	**PG**	**PSG**	**PG**
**STOP-BANG ≥3**	**Sensitivity**[P]	97.12	95.24	98.52	98.18	99.46	99.15
	**Specificity**[P]	30.39	28.42	15.45	18.7	10.46	12.93
	**AUC**[P]	0.638	0.618	0.570	0.584	0.550	0.560
**STOP-BANG ≥5**	**Sensitivity**[P]	67.94	66.4	73.65	75.14	80.14	80.15
	**Specificity**[P]	74.13	75.96	59.41	64.89	51.45	53.21
	**AUC**[P]	0.710	0.712	0.665	0.700	0.658	0.667

Abbreviation: AHI, apnea-hypopnea index; AUC, *area under the curve; PG, polygraphy; PSG, polysomnography*

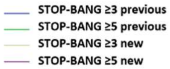

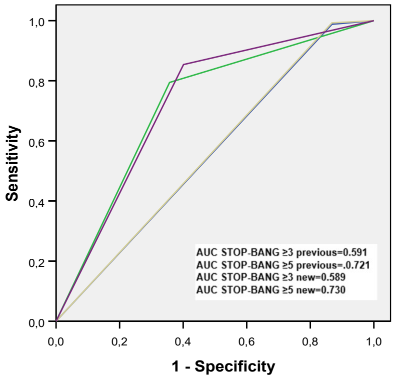

AHI≥5 events/h Greece

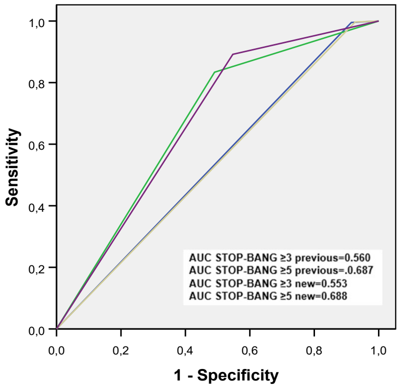

AHI≥15 events/h Greece

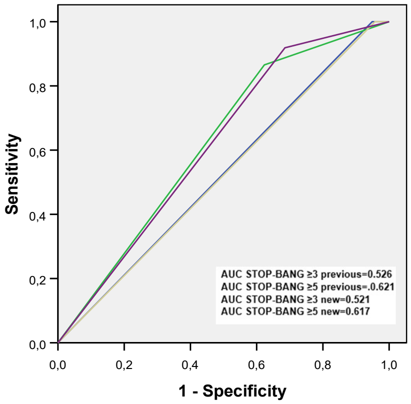

AHI≥30 events/h Greece

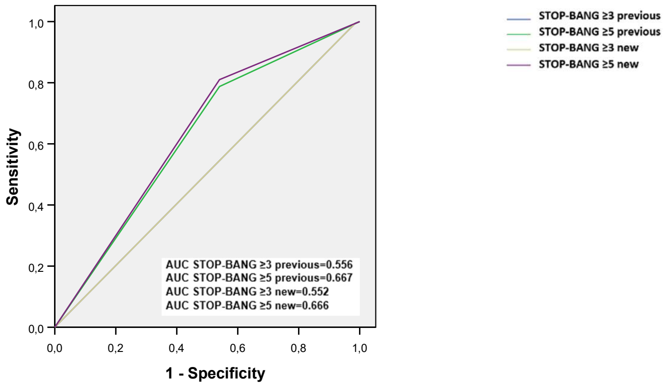

AHI≥5 events/h Türkiye

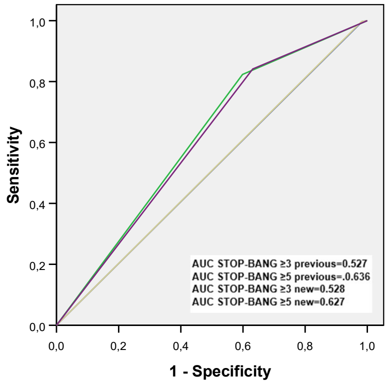

AHI≥15 events/h Türkiye

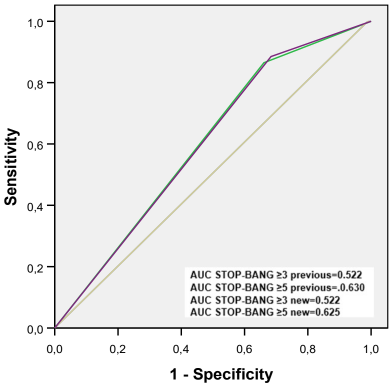

AHI≥30 events/h Türkiye

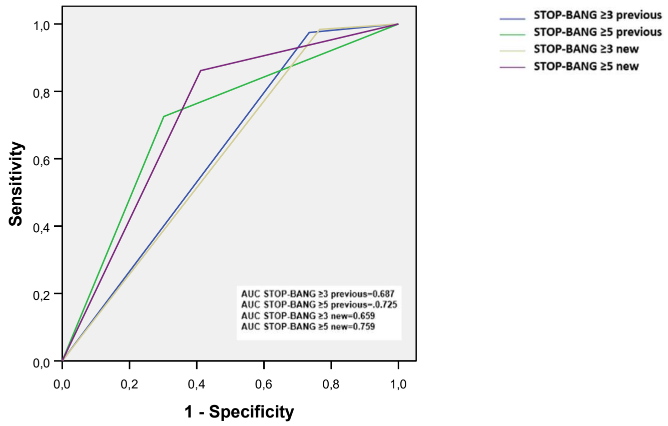

AHI≥5 events/h Croatia

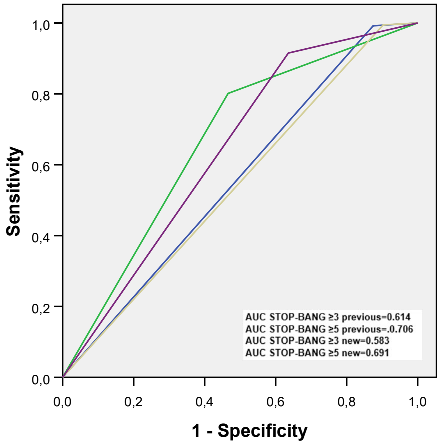

AHI≥15 events/h Croatia

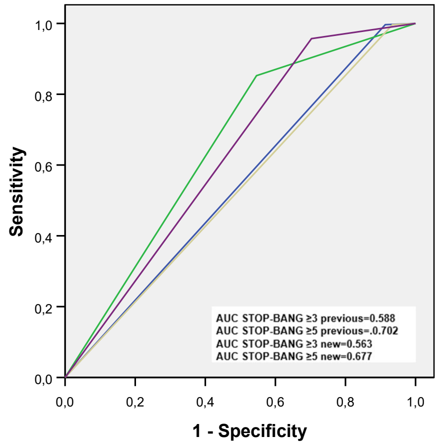

AHI≥30 events/h Croatia

Figure S1. Receiver operating characteristic (ROC) curve figures illustrating the area under the curve (AUC) values for the STOP and STOP-BANG questionnaire approaches, presenting the comparative screening performance for detecting obstructive sleep apnea at different apnea-hypopnea index (AHI) thresholds across the studied populations.
